# Inflammatory Response and Chemokine Expression in the White Matter Corpus Callosum and Gray Matter Cortex Region During Cuprizone-Induced Demyelination

**DOI:** 10.1007/s12031-012-9773-x

**Published:** 2012-04-22

**Authors:** J. P. Buschmann, K. Berger, H. Awad, T. Clarner, C. Beyer, M. Kipp

**Affiliations:** 1 Institute of Neuroanatomy, Faculty of Medicine, RWTH Aachen University, Wendlingweg 2, 52074 Aachen, Germany; 2 Department of Pathology, VU University Medical Center, De Boelelaan 1117, 1081 HV Amsterdam, the Netherlands; 3 RWTH Aachen, Institute of Neuroanatomy, Wendlingweg 2, 52074 Aachen, Germany

**Keywords:** Cuprizone, CNS, Inflammation, Chemokines, Demyelination, Multiple sclerosis

## Abstract

Brain inflammation plays a central role in multiple sclerosis (MS). Besides lymphocytes, the astroglia and microglia mainly contribute to the cellular composition of the inflammatory infiltrate in MS lesions. Several studies were able to demonstrate that cortical lesions are characterized by lower levels of inflammatory cells among activated microglia/macrophages. The underlying mechanisms for this difference, however, remain to be clarified. In the current study, we compared the kinetics and extent of microglia and astrocyte activation during early and late cuprizone-induced demyelination in the white matter tract corpus callosum and the telencephalic gray matter. Cellular parameters were related to the expression profiles of the chemokines *Ccl2* and *Ccl3*. We are clearly able to demonstrate that both regions are characterized by early oligodendrocyte stress/apoptosis with concomitant microglia activation and delayed astrocytosis. The extent of microgliosis/astrocytosis appeared to be greater in the subcortical white matter tract corpus callosum compared to the gray matter cortex region. The same holds true for the expression of the key chemokines *Ccl2* and *Ccl3*. The current study defines a model to study early microglia activation and to investigate differences in the neuroinflammatory response of white vs. gray matter.

## Introduction

Multiple sclerosis (MS), a chronic inflammatory and demyelinating disease, was first identified as a separate neurological disorder by the French neurologist Jean-Martin Charcot and is a common disorder of the central nervous system (CNS) in young adults. Postmortem tissue is widely used to study which molecular and cellular mechanisms are involved in MS lesion development. The histopathological hallmarks of the lesions are inflammation, demyelination, and axonal degeneration (van der Valk and De Groot 2000; Frischer et al. 2009). Different types of lesions have been classified on the histological level, the active ones containing a high number of microglia/macrophages (Barnett and Prineas 2004; Bitsch et al. 2001). Using general histological methods such as hematoxylin and eosin (H&E) or LFB stain in combination with immunohistochemistry (IHC) for myelin and human leukocyte antigen (HLA)-DR antigens, lesions can be classified into several stages. Pre-active lesions contain activated microglial cells (strongly positive for HLA-DR); however, there is no overt demyelination (van der Valk and Amor 2009). Active demyelinating lesions are hypercellular due to the macrophages and/or microglia which are phagocytosing myelin sheaths, giving them a so-called foamy appearance. Chronic active lesions contain a hypocellular center and a hypercellular rim with myelin-containing macrophages, indicating ongoing demyelination at the lesion edge and complete demyelination with cessation of inflammatory processes in the center of the lesion. Finally, chronic inactive lesions are hypocellular, although some residual inflammatory activity might be present at the borders of the lesion (van der Valk and De Groot 2000; van der Valk and Amor 2009). It has been recently reported that pronounced inflammation in the brain is present not only in acute and relapsing MS but also in the secondary and primary progressive disease (Frischer et al. 2009). Thus, brain inflammation plays a critical role in MS pathogenesis and disease progression.

Two categories of molecules direct leukocyte migration and microglia activation into inflammatory sites: adhesion molecules and cytokines. Among cytokines, chemokines (small, pro-inflammatory chemotactic cytokines) have gained particular interest because of their potential role in pathogenic inflammation (Luster 1998; Baggiolini 1998). Chemokines possess the ability to induce chemotaxis (directional migration) and non-directional cell migration of a variety of cell types, particularly leukocytes (Karpus and Ransohoff 1998; Sozzani et al. 1996). Furthermore, chemokines have been shown to be important for cell migration in the absence of inflammation (Patel et al. 2010). Indeed, chemokines now appear to have multiple biological activities in addition to their known role in mobilizing inflammatory cells. Their expression is induced in various pathologies of the CNS, among Alzheimer’s disease, Behçet’s disease, HIV-1-associated dementia, human T cell leukemia virus-1-associated myelopathy, or brain injury (McMahon et al. 2001; Glabinski et al. 1997). In particular, chemokines are induced in the demyelinating disease MS and numerous animal models for demyelination, including experimental autoimmune encephalomyelitis (EAE), Theiler’s and hepatitis virus-induced demyelinating disease, experimental autoimmune neuritis, and twitcher, a murine model of globoid cell leukodystrophy (McMahon et al. 2001).

Four subfamilies of chemokines have been classified—C(γ), CC(β), CXC(α), or CX3C(δ)—according to the number and spacing between the cysteine residues in the N-terminal region. They signal via seven G-protein-coupled transmembrane receptors of the serpentine superfamily (Hoffman et al. 2001). Most chemokines display affinity for more than one receptor and elicit biological activities at nanomolar concentrations in vivo. Differential expression of a number of chemokines and their receptors has been demonstrated in both acute and chronic MS lesions, including monocyte chemotactic protein-1 (MCP-1 or CCL2) and macrophage inflammatory protein-1α (MIP1α or CCL3; Szczucinski and Losy 2007). Through construction of transgenic and knockout mice, much progress has been made in our understanding of chemokine action in vivo. Several research groups have used EAE to probe CNS chemokine expression and function. Karpus and colleagues showed that anti-CCL3 antibodies suppress the initial attack of adoptive-transfer EAE, while anti-CCL2 antibodies inhibit subsequent relapses, indicating that specific chemokines execute non-redundant functions in these disease models (Karpus and Ransohoff 1998; Karpus et al. 1995; Karpus and Kennedy 1997).

In MS, brain inflammation is associated with lesions appearing typically in plaques within the white matter. Histopathological studies, however, now convincingly show that gray matter regions, such as the forebrain cortex (Cx), are also affected (Geurts and Barkhof 2008; Geurts et al. 2007; Lucchinetti et al. 2011). The pathology of gray matter lesions differs from white matter lesions in several aspects, such as less pronounced lymphocyte infiltration, macrophage activity, complement composition, or blood–brain barrier (BBB) alterations (Bo et al. 2003; Breij et al. 2008; van Horssen et al. 2007; Peterson et al. 2001). The observed differences could be due to the differences in the composition and function of the BBB or other elements of the cytoarchitecture of the cerebral Cx, which makes it more resistant to inflammation (Bo et al. 2003). Given the extensive cortical involvement in MS patients, the low T and B cell infiltration in cortical lesions, and the extensive white matter demyelination in most MS patients, it has been proposed that cortical lesions are degenerative in origin. However, whether or not the development of cortical demyelinated lesions in MS displays a separate identity remains to be clarified. Comparative analyses are warranted to detect not only differences but also similarities in the genesis of white vs. gray matter lesions in MS patients.

As pointed out above, chemokines appear to be critically involved in the induction of an inflammatory response during MS lesion formation. The purpose of this study was to compare the kinetics and extent of microglia and astrocyte activation during cuprizone-induced demyelination in the white matter tract corpus callosum and the telencephalic gray matter. Cellular parameters were related to the expression profile of the chemokines *Ccl2* and *Ccl3* to detect a relationship of neuroinflammatory responses and local chemokine levels.

## Material and Methods

### Animals and Demyelination

C57BL/6J mice were obtained from Charles River (Germany) and underwent routine cage maintenance once a week and microbiological monitoring according to the Federation of European Laboratory Animal Science Association’s recommendations. Food and water were available ad libitum. Animal care procedures were approved by the Review Board for the Care of Animal Subjects of the district government (Nordrhein-Westfalen, Germany) and performed according to international guidelines on the use of laboratory animals. Demyelination was induced by feeding 8- to 9-week-old (19–21 g) male mice a diet containing 0.2 % cuprizone (bis-cyclohexanone oxaldihydrazone, Sigma-Aldrich Inc., Germany), mixed into a ground standard rodent chow for the indicated period as previously described (Clarner et al. 2011; Acs et al. 2009; Kipp et al. 2011a). Control animals were fed normal powdered chow.

### Tissue Preparation

Tissue preparation was performed as previously described (Acs et al. 2009; Groebe et al. 2009; Kipp et al. 2008). For histological and immunohistochemical studies, mice were transcardially perfused with 2 % paraformaldehyde containing picric acid. After overnight post-fixation, brains were dissected, embedded, and then coronary processed into 5-μm sections from the levels 215 to 275 according to the mouse brain atlas by Sidman et al. (http://www.hms.harvard.edu/research/brain/atlas.html). For gene expression analysis, mice were transcardially perfused with ice-cold PBS, the brains quickly removed, and the entire corpus callosum without the adjacent cortex dissected using a stereomicroscopic approach. Isolated corpus callosi and cortices were snap-frozen in liquid nitrogen and kept at −80°C until used.

### IHC and Cell Parameter Quantification

For IHC, sections were placed on silane-coated slides, deparaffinized, rehydrated, heat-unmasked, blocked with PBS containing 5 % normal serum, and incubated overnight with the primary antibody diluted in blocking solution. Anti-proteolipid protein antibody (PLP, 1:500, mouse IgG; Serotec, Germany) was used as a myelin marker; anti-adenomatous polyposis coli (APC, 1:200, mouse IgG; Calbiochem, Germany) was used to stain late-stage oligodendrocyte cell bodies, while anti-glial fibrillary acidic protein (GFAP, 1:1,000, rabbit IgG; Encore, USA) was used to visualize astrocytes. Anti-ionized calcium-binding adaptor molecule antibody (IBA1, 1:250, rabbit IgG; Wako, Germany) was used as a marker to detect microglia/macrophages. An antibody against 2′3′-cyclic nucleotide 3′-phosphodiesterase [anti-CNPase (SMI-91) mouse monoclonal IgG1, Abcam, UK) was used to detect oligodendrocytes and myelin fibers and an anti-active caspase 3 antibody (anti-active caspase 3, 1:250, rabbit IgG; Abcam) was used to visualize apoptotic cells (Acs et al. 2009; Kipp et al. 2011a; Norkute et al. 2009; Dang et al. 2011). After washing and blocking of endogenous peroxidase with hydrogen peroxide (0.3 %, 30 min), sections were incubated with biotinylated secondary anti-mouse or anti-rabbit IgG antibodies (1:50; Vector Laboratories, Burlingame, CA, USA) for 1 h, followed by peroxidase-coupled avidin–biotin complex (ABC kit, Vector Laboratories). The immunoprecipitated product was visualized with the AEC or DAB reaction (Invitrogen, Darmstadt, Germany). For immunofluorescence staining, Alexa Fluor 488 and/or Alexa Fluor 568 (both 1:500, Invitrogen) were used and controls performed as published (Baertling et al. 2010). Standard H&E stain was performed on deparaffinized sections for the evaluation of apoptosis using well-defined morphological criteria, such as condensed and fragmented cell nuclei (Hesse et al. 2010; Kipp et al. 2011b). Subsequently, stained and processed sections were documented with a Nikon ECLIPSE 80i microscope. Fluorescence microscopy was performed using the microscope working station Zeiss LSM 7 Duo (Zeiss, Germany).

Counting was performed by two independent readers blinded for treatment groups; the results were averaged. Cell numbers are given in cells per square millimeter. For the corpus callosum, cell parameters were quantified in both the medial and lateral parts and the values averaged. For the cortex, cell parameters were quantified on the same level in the outer (layers 1–3) and inner parts (layers 4–6) and the values averaged. Determination of cell area was performed using NIS Elements 3.0 software (Nikon Corp., Japan). Cell areas were measured using the auto-detect area tool. Area was determined for 15 cells in each indicated region.

### Real-Time Reverse Transcriptase Polymerase Chain Reaction

Gene expression levels were determined using the rt-PCR technology (BioRad, Germany), QTM SYBR Green Supermix (Quantace, Germany), and a standardized protocol as described previously (Kipp et al. 2011a; Groebe et al. 2009; Braun et al. 2009). Isolation of total RNA was performed using the NucleoSpin kit (Macherey-Nagel, Germany). RNA was reverse-transcribed using an M-MLV RT kit and random hexanucleotide primers (both Invitrogen). PCR reactions were carried out in a reaction mixture consisting of 2 μl cDNA, 2 μl RNAse-free water, 5 μl QTM SYBR Green Supermix, and 0.5 μl of each primer (10 pmol/μl) in standard 96-well plates under the following conditions: 10 min enzyme activation at 95°C, 40 cycles of 15 s denaturation at 95°C, 30 s annealing at individual temperatures, 30 s amplification at 72°C, and 5 s fluorescence measurement at 80°C. Primer sequences are given in Table 1. Melting curves and gel electrophoresis of the PCR products were routinely performed to determine the specificity of the PCR reaction (not shown).

**Table 1:** Primer sequences

Primer	Sequence	Length (bp)	AT (°C)
*18S*	cggctaccacatccaaggaa	187	60
	gctggaattaccgcggct		
*Gfap*	cagatgatggagctcaatgacc	379	60
	ctggatctcctcctccagcga		
*Plp*	tggcgactacaagaccacca	115	60
	gacacacccgctccaaagaa		
*Ccl2*	ttaaaaacctggatcggaaccaa	120	60
	gcattagcttcagatttacgggt		
*Ccl3*	ttctctgtaccatgacactctgc	99	62
	cgtggaatcttccggctgtag		

Relative quantification was performed by comparing the *C*_t_ values of samples with the *C*_t_ values of an internal standard curve, ranging from 100 % (undiluted) to 1.56 % (seven times, 1:1 dilution). The results were normalized using the Δ*C*_t_ method, which results in ratios between the target genes and a housekeeping reference gene (*18s*). As the validity of this method critically depends on the constant expression of the housekeeping gene, constant expression of *18s* was verified (not shown).

To calculate the semiquantitative expression ratios between the corpus callosum and cortex, additional cDNA was generated by reverse transcription of the control group samples from the corpus callosum and the cortex in one reverse transcription reaction. After subsequent PCR analysis of *18s* expression in these samples, differences in the expression between the corpus callosum and the cortex in control animals were calculated for *Ccl2* and *Ccl3*. This ΔΔ*C*_t_ calculation was done using REST-2009 software (Pfaffl et al. 2002). Knowing the differences between the controls enabled us to calculate expression differences for the 1-week samples since the expression data for each region are expressed relative to the control.

### Statistical Analysis

Data represent the results of at least two independent experiments. Statistics were made using absolute data of all experiments. For IHC as well as rt-PCR, differences between groups were tested using ANOVA followed by Tukey’s post hoc test. GraphPad Prism 5 (GraphPad Software Inc.) was used. The statistical significance of the difference of each group compared to the control is indicated (*p ≤ 0.05; **p ≤ 0.01; ***p ≤ 0.001). All data are given as arithmetic means ± SEM. At least three mice each for IHC and four mice each for gene expression analysis were used per experimental group.

## Results

### Oligodendrocyte Loss Starts Early in the Cuprizone Model

Cuprizone ingestion in mice induces a highly reproducible demyelination of distinct brain regions, among them the white matter tract corpus callosum (CC) and the telencephalic gray matter Cx region (Kipp et al. 2009; Skripuletz et al. 2008). In a first set of experiments, we aimed to investigate the temporal dynamics of oligodendrocyte loss, demyelination, microglia activation, and astrocytosis during cuprizone intoxication. Mice were fed up to 5 weeks cuprizone and killed at half a week (i.e., 2 days), weeks 1, 2, 3, and 5 after initiation of the cuprizone diet. Brain sections were immunohistochemically stained for the myelin marker protein PLP, the oligodendrocyte cell body marker APC, the (activated) astrocyte-specific protein GFAP, and the microglia/macrophage marker protein IBA1.

**Fig. 1:**
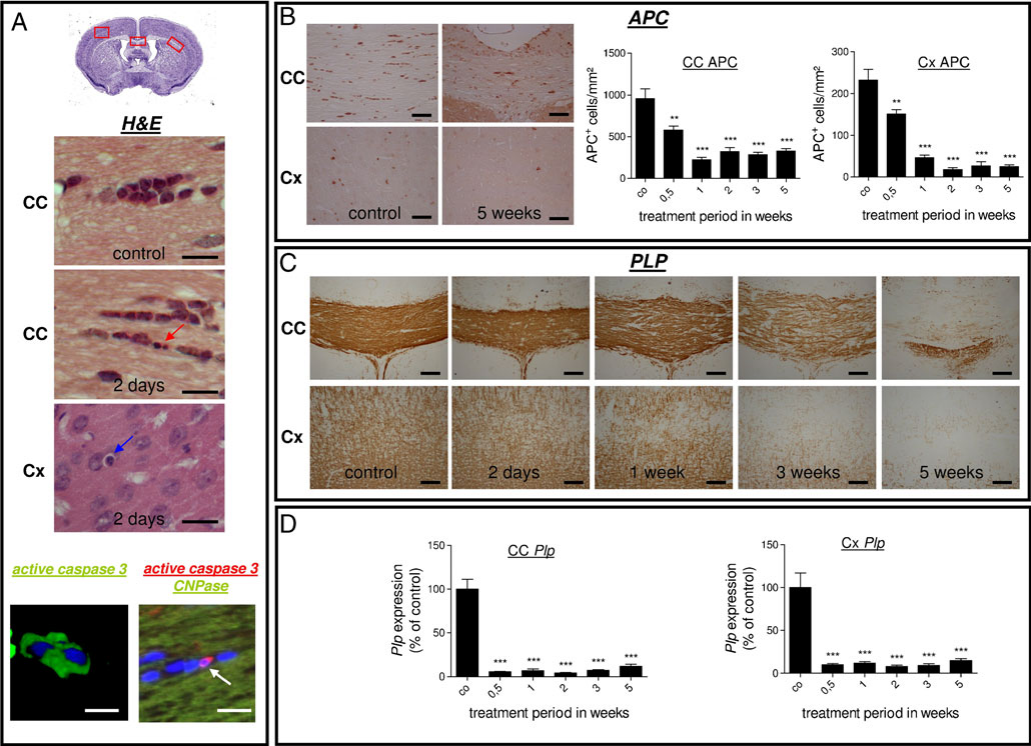
**a** HE-stained sections of the midline of the corpus callosum in control and 2 days cuprizone-treated animals as well as a 2-day cortex sample. On day 2 after the start of the cuprizone diet, cells with the typical morphological characteristics of apoptosis, such as round, condensed, and fragmented nuclei, were detected in both regions (*red* and *blue arrows*). The apoptotic nature was further verified by anti-caspase 3 staining (*lower left*) and anti-caspase 3/CNPase double labeling (*lower right*). **b** *Left part* Anti-APC stained sections of the midline of the corpus callosum as well as cortex regions of a control animal and after 5 weeks of cuprizone exposure. *Right part* Results of APC-expressing cell quantification within the corpus callosum and cortex regions. **c** Anti-PLP-stained sections of the midline of the corpus callosum and cortex regions in control and 2 days and 1, 3, and 5 weeks cuprizone-treated animals. Note that no overt loss of PLP staining intensity can be observed until week 1 in both regions included in the study, whereas the midline of the corpus callosum and the cortex regions are significantly demyelinated at week 3. Demyelination further progresses until week 5. **d** Results of *Plp* gene expression analysis of the entire corpus callosum or cortex region. Each *bar* represents the averaged fold induction over untreated control mice of at least four mice per time point (±SEM). Values were normalized against a housekeeping gene (*18s*) and expressed relative to the respective control levels. *p ≤ 0.05; **p ≤ 0.01; ***p ≤ 0.001 treatment vs. control. *Scale bars*, 15 μm (*upper part* in **a**); 10 μm (*lower left* in **a**); 5 μm (*lower right* in **a**); 50 μm (**b**); and 80 μm (**c**)

Regions included in the study are the CC (medial and lateral parts) and the somatosensory Cx region (areas highlighted in Fig. 1a by red inserts). As demonstrated, oligodendrocyte death starts early after initiation of the cuprizone diet. Numerous apoptotic oligodendrocytes (i.e., condensed and/or fragmented nuclei of cells in a chain-like formation; Fig. 1a, red arrow) were seen after 2 days in the CC of cuprizone-fed animals, whereas myelination, as determined by anti-PLP staining, was still normal (see Fig. 1c). The apoptotic nature of these cells was verified by anti-active caspase 3 staining (Fig. 1a, lower left part). To further confirm that oligodendrocytes undergo apoptotic cell death, we stained for activated caspase 3 combined with immunohistochemistry for CNPase. As demonstrated, activated caspase 3-expressing cells can be found in close vicinity to CNPase-reactive fibers (Fig. 1a, lower right, white arrow). Not only in the CC but also in the Cx region were apoptotic cells observed, although in reduced numbers as compared to the white matter tract CC. Furthermore, the morphology of dying oligodendrocytes in the Cx region was slightly different. The nuclei were condensed, but only rarely fragmented nuclei were observed (Fig. 1a, blue arrow). Control animals were lacking those cells. In parallel, we found a marked decrease in the number of APC^+^ oligodendrocytes in both regions (Fig. 1b). Already after 2 days of cuprizone intoxication, the numbers of APC^+^ mature oligodendrocytes were significantly decreased in both regions included in the study. Myelination, as determined by anti-PLP IHC, was still normal at weeks 1–2. At week 3, however, both regions displayed significant loss of anti-PLP immunoreactivity. At week 5, both regions were almost completely devoid of PLP-reactive fibers (Fig. 1c). Furthermore, we investigated the effects of cuprizone treatment on PLP gene expression separately for both regions. As shown in Fig. 1d, *Plp* mRNA expression levels showed a marked reduction already after 2 days in both regions and remained at these low levels during the entire investigation period. The observed early decline of PLP mRNA species suggests an active down-regulation of oligodendrocyte-related gene transcription.

### Microglia Activation Is Evident Before Overt Demyelination

**Fig. 2:**
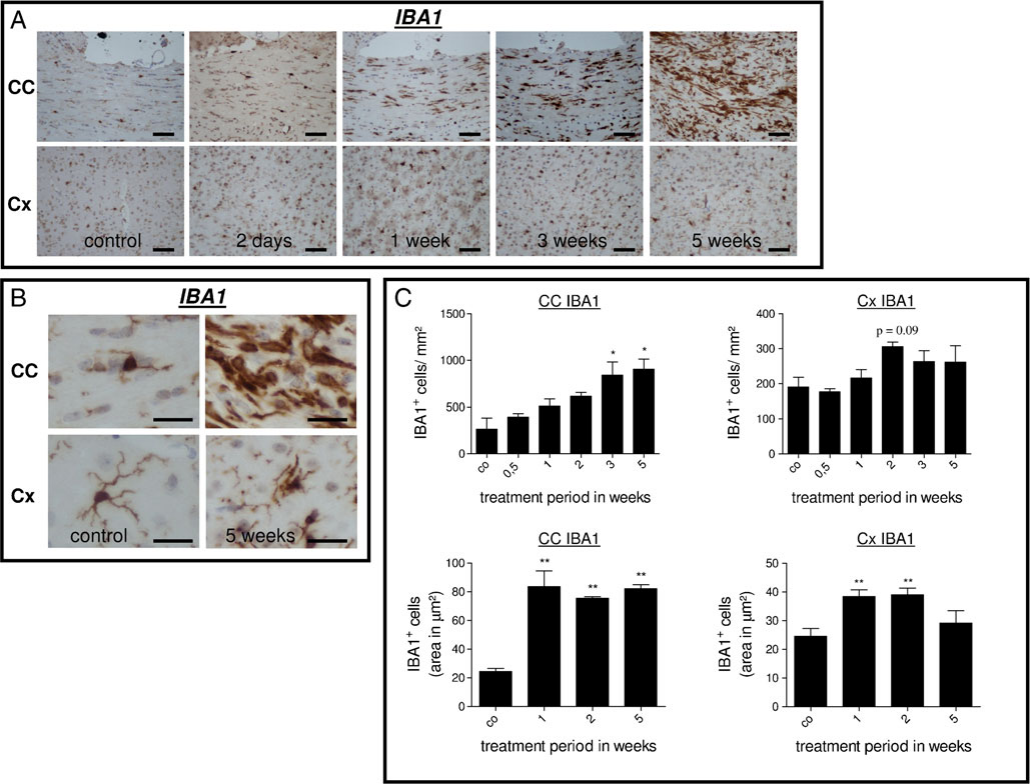
**a** Anti-IBA1-stained sections of the midline of the corpus callosum and cortex regions in control and 2 days and 1, 3, and 5 weeks cuprizone-treated animals. Note the early increase in microglia cell numbers at day 2 and the pronounced increase at week 5, respectively. **b** Higher magnification micrographs of IBA1-positive cells within the midline of the corpus callosum and cortex regions in control animals and after 5 weeks cuprizone treatment. Note the pronounced increase in number and size after 5 weeks within the midline of the corpus callosum. **c** *Upper row* Results of IBA1-expressing cell quantification within the corpus callosum and cortex regions. *Lower row* Morphometric quantification of IBA1-expressing cell area within the corpus callosum and cortex regions. Note that microglia hyperplasia and hypertrophy are more pronounced in the affected white matter corpus callosum compared to the gray matter cortex region. *p ≤ 0.05; **p ≤ 0.01; ***p ≤ 0.001 treatment vs. control. *Scale bars*, 50 μm (**a**) and 15 μm (**b**)

In the cuprizone model, loss of oligodendrocytes involves the expansion and activation of microglia cells and astrocytes (Kipp et al. 2009, 2011a, b). In a next step, we aimed to characterize the extent and dynamics of microglia activation during the early and late stages of cuprizone-induced demyelination. To this end, we quantified the number of microglia cells in IBA1-stained sections in the CC and Cx. Furthermore, the mean microglia volume was estimated by measuring the surface area covered by IBA1-expressing cells. As demonstrated in Fig. 2, we observed an increase in IBA1^+^ cell numbers in the CC after the earliest time point included in the study (2 days). Thereafter, the number of microglia steadily increased until week 5. Microgliosis was less pronounced and delayed in the Cx. Furthermore, a marked hypertrophy of IBA1^+^ cells was evident in the CC after 1 week of cuprizone treatment. The increase in microglia cell size was by far less distinctive in the cortical gray matter (Fig. 2c, lower part).

**Fig. 3:**
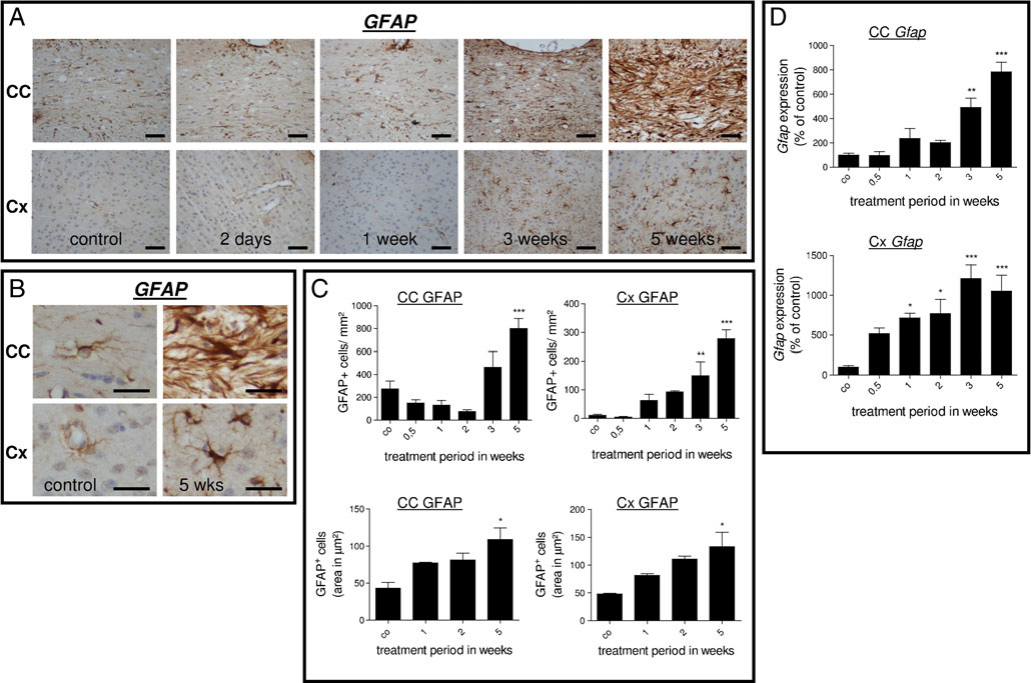
**a** *Upper row* Anti-GFAP-stained sections of the midline of the corpus callosum in control and 2 days and 1, 3, and 5 weeks cuprizone-treated animals. Note the severe astrocytosis at week 5. *Lower row* Anti-GFAP-stained sections of the cortex in control and 2 days and 1, 3, and 5 weeks cuprizone-treated animals. Note the moderate increase in GFAP^+^ cell numbers at week 5. **b** Micrographs in higher magnification of GFAP^+^ cells within the midline of the corpus callosum and cortex regions in control animals and after 5 weeks cuprizone treatment. Note the pronounced increase in number and size after 5 weeks within the midline of the corpus callosum. **c** *Upper row* Results of GFAP-expressing cell quantification within the corpus callosum and cortex regions. *Lower row* Morphometric quantification of GFAP-expressing cell area within the corpus callosum and cortex regions. Note that the extent of astrocyte hypertrophy is comparable in the affected white matter corpus callosum and the gray matter cortex region. **d** Results of *Gfap* gene expression analysis of the entire corpus callosum or cortex region. Each *bar* represents the averaged fold induction over untreated control mice of at least four mice per time point (±SEM). *p ≤ 0.05; **p ≤ 0.01; ***p ≤ 0.001 treatment vs. control. *Scale bars*, 50 μm (**a**) and 15 μm (**b**)

The same cellular parameters were determined for GFAP-expressing cells to assess the extent of astrocytosis. The number of GFAP^+^ astrocytes was higher in the CC compared to the Cx in control animals (Fig. 3a, c). During cuprizone-induced demyelination, GFAP^+^ cell numbers increased in both regions. Absolute numbers of GFAP-expressing astrocytes in the CC exceeded those in the Cx at any time point included in the study. Astroglia volume was equal in the CC and Cx regions in untreated controls and evenly increased during the course of cuprizone-induced demyelination (Fig. 3c, lower part). Furthermore, we investigated the effects of cuprizone treatment on *Gfap* gene expression (Fig. 3d). Within the CC, *Gfap* mRNA expression was significantly up-regulated at weeks 3 and 5 compared to the controls. A significant induction of *Gfap* mRNA expression levels within the Cx was evident already after week 1.

In summary, both regions display severe early oligodendrocyte cell loss, isochronous microgliosis, followed by astroglia activation and myelin loss. The extent of the brain-intrinsic cellular inflammatory response is by far greater within the affected white matter tract CC compared to the demyelinated cortex region. Furthermore, microgliosis in the white matter CC is clearly evident at week 1, while myelination is still normal (Figs. 1c and 2a, c).

### *Ccl2* (Mcp1) and *Ccl3* (Mip1α) Expression Up-regulation Occurs Early in Cuprizone-Induced Demyelination

**Fig. 4:**
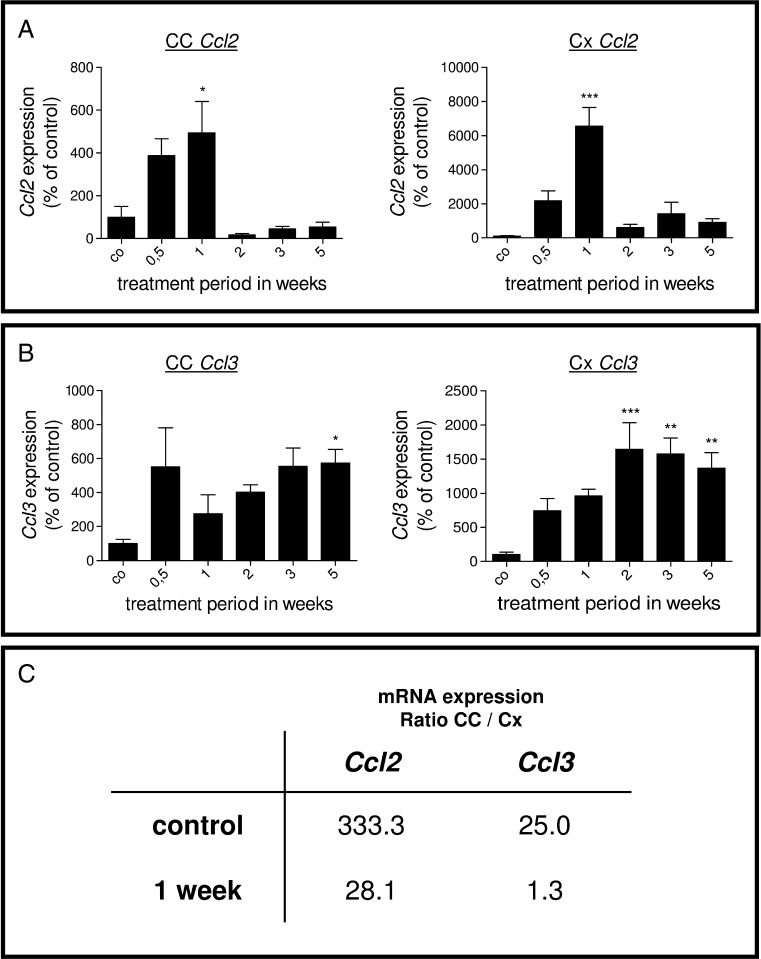
**a**, **b** Results of *Ccl2* (**a**) and *Ccl3* (**b**) gene expression analysis by rt-PCR of the entire corpus callosum or cortex region. Each *bar* represents the averaged fold induction over untreated control mice of at least four mice per time point (±SEM). Values were normalized against a housekeeping gene (*18s*) and expressed relative to the respective control levels. Note the early induction of *Ccl2* and *Ccl3* mRNA expression levels. **c** mRNA expression ratio CC vs. Cx in control animals and after 1 week cuprizone exposure for *Ccl2* and *Ccl3*. Note that the basal mRNA levels of *Ccl2* are 333.3 times greater and those of *Ccl3* are 25 times greater in the corpus callosum compared to the cortex region (both *p* ≤ 0.001). *p ≤ 0.05; **p ≤ 0.01; ***p ≤ 0.001 treatment vs. control

CCL2 and CCL3 are among the best characterized chemokines. Previous studies revealed that both peptides might be involved in MS pathology (McManus et al. 1998; Okada et al. 2010). Since studies of the cellular infiltrate displayed that the dynamics and extent of microgliosis at the site of lesion differs between the white and gray matter, we speculated that the induction of chemokine expression displays a region-specific kinetic/magnitude, as well. Therefore, rt-PCR analyses for *Ccl2* and *Ccl3* were separately performed in dissected CC and Cx samples during a 5-week cuprizone exposure period. During cuprizone-induced demyelination, two expression profiles were evident. *Ccl2* mRNA expression in CC and the Cx peaked at week 1, but thereafter appeared to be reduced (Fig. 4a). In contrast, *Ccl3* mRNA expression gradually increased in both regions (Fig. 4b). The extent of chemokine expression induction was by far greater within the Cx compared to the CC. Figure 4c displays a comparative evaluation of the expression levels between the CC and Cx regions at a given time point (i.e., control and 1 week). This analysis revealed that the basal expression of *Ccl3* is 25 times higher in the CC compared to the Cx. For *Ccl2*, the mRNA levels are 333.3 times higher in the CC compared to the Cx. At week 1, *Ccl3* mRNA levels are 1.3 times higher in the CC compared to the Cx. For Ccl2, the mRNA levels are 28.1 times higher in the CC compared to the Cx at week 1.

## Discussion

In this study, we were able to demonstrate that cuprizone treatment affects oligodendrocyte viability and, in consequence, myelin integrity in the white matter tract CC and the telencephalic gray matter region. These results are in line with previously reported findings. Skripuletz et al. (2008) were the first to demonstrate that in addition to the expected demyelination in the CC, the cortex of C57BL/6 mice is completely demyelinated after acute cuprizone-induced demyelination. In line with their findings, numbers of IBA1-expressing microglia were not significantly increased in the affected cortex region. However, we are now able to demonstrate by means of morphometric estimation of the microglia volume that this cell population is indeed activated in both regions, although an increase in microglia cell size was by far less distinctive in the cortical gray matter compared to the white matter tract CC.

In MS, the pathology of gray matter lesions is believed to differ from white matter lesions in several aspects, such as less pronounced lymphocyte infiltration, macrophage activity, complement composition, or BBB alterations (Bo et al. 2003; Breij et al. 2008; van Horssen et al. 2007; Peterson et al. 2001). The observed differences could be due to the differences in the composition and function of the BBB or other elements of the cytoarchitecture of the cerebral Cx, which makes it more resistant to inflammation (Bo et al. 2003). Whether or not the development of cortical demyelinated lesions in MS displays a separate identity remains to be clarified. In the cuprizone model, one would speculate that the underlying mechanism of oligodendrocyte pathology does not differ between both regions. The lower magnitude of microglia activation within the gray matter cortex might be due to the differences in the cortical cytoarchitecture. However, the reduced inflammatory response in cortical lesions, especially microglia/macrophage activation, could be simply due to the lower cortical myelin content and, in consequence, less myelin debris during a demyelinating event. The same might be true for less pronounced astrogliosis; however, systematic studies addressing this issue were not yet performed.

Another important finding of this study was that in the toxic demyelination cuprizone model, oligodendroglial cell death and a reduction of myelin gene expression levels start days after the initiation of the cuprizone diet and weeks before demyelination is evident. This observation is in line with a study of Hesse et al. (2010) who were further able to demonstrate that in the early but not in the later stages, dying oligodendrocytes express activated caspase 3, suggesting a switch from classical apoptotic pathways to caspase 3-independent mechanisms during the course of the cuprizone diet. We are the first to demonstrate that early oligodendrocyte loss is not only restricted to the CC but can also be observed in the cortex region. Both the number of APC-expressing oligodendrocytes as well as the *Plp* mRNA levels are dramatically reduced in both regions included in this study at week 1. The phenomenon of oligodendrocyte apoptosis/stress and microglia activation in a normally myelinated tissue (at least if investigated by conventional immunohistochemistry) has also been described in MS tissue. In 2001, the Amsterdam Neuropathology Group described the selection of MS lesions for further characterization using a novel MRI-guided sampling protocol (De Groot et al. 2001). This novel protocol revealed lesion-like signals on MRI scans that were not the traditional MS lesions upon routine histological examination. Subsequent histological analysis of these lesions has led to the realization that the abnormalities seen by MRI correlated with the well-circumscribed clusters of activated microglial cells, notably in the absence of demyelination, i.e., in otherwise normal-appearing white matter. The activated state of the microglia was reflected by the increased expression of HLA-DR and CD68. The authors termed the observed abnormalities “pre-active” lesions due to the assumption that they precede classical active demyelinating lesions, at least in a subset of patients (van der Valk and De Groot 2000; van der Valk and Amor 2009; van Noort et al. 2011). The general awareness of pre-active lesions as a real phenomenon in MS is surprisingly limited. This may have something to do with the fact that the several studies that have documented them in great detail all use different terms to describe them. The first study called them “type I lesions” (Gay 2006), the second “(p)re-active lesions” (De Groot et al. 2001), and the third “newly forming lesions” (Barnett and Prineas 2004), while more recently they were called “pattern III lesions” (Marik et al. 2007). Yet, they all refer to the same phenomenon. We speculate that short-term cuprizone exposure models very well such early pathological stages during MS lesion formation (Kipp et al. 2011b), and analyzing the affected CC in detail might help in understanding the underlying mechanisms and the relevance of early microglia activation in MS pathogenesis and disease progression.

CCL2 (MCP1) and CCL3 (MIP-1α) are among the best characterized chemokines. Previous studies revealed that both chemokines might be involved in MS pathology. For example, in experimental autoimmune neuritis, administration of anti-CCL2 antibodies delayed the onset of the disease (Zou et al. 1999). Anti-CCL2 significantly reduced the severity of relapsing EAE (Karpus and Kennedy 1997). CCL3 expression correlated with acute disease development, whereas CCL2 did not. In contrast, CCL2 production in the CNS correlated with relapsing EAE development. Moreover, anti-CCL3 inhibited the development of acute but not relapsing EAE. During this study, analyses of chemokine gene expression levels revealed that *Ccl2* and *Ccl3* mRNA levels significantly increased in both regions. Interestingly, *Ccl2* induction was a transient phenomenon, whereas the mRNA levels for *Ccl3* continuously increased. One might speculate that CCL2 mediates early microglia activation, whereas the function of CCL3 orchestrates later cellular events such as the induction of astrocyte activation and/or sustenance of microgliosis. Remarkably, comparative analysis revealed that *Ccl2* and, although to a lesser extent, *Ccl3* levels are, at least on the mRNA level, greater in the CC compared to the cortex region. This finding strongly suggests that both chemokines somehow mediate lower inflammatory infiltrates in demyelinated gray matter lesions. Further studies have to show the functional relevance of early CCL2 expression in this animal model, the impact of sustained CCL3 levels on astrocyte activation and maybe myelin repair, which is evident in the later stages (Kipp et al. 2009, 2011a, c), and the source of respective chemokines on the cellular level.

## Abbreviations

APC: Adenomatous polyposis coli
BBB: Blood–brain barrier
CC: Corpus callosum
CNPase: 2′3′-Cyclic nucleotide 3′-phosphodiesterase
CNS: Central nervous system
Cx: Cortex
EAE: Experimental autoimmune encephalomyelitis
GFAP: Glial fibrillary acidic protein
H&E: Hematoxylin and eosin stain
HLA: Human leukocyte antigen
LFB: Kluver–Barrera/luxol fast blue stain
MCP-1: Monocyte chemotactic protein-1 or CCL2
MIP1α: Macrophage inflammatory protein-1α or CCL3
MS: Multiple sclerosis
PLP: Proteolipid protein
